# In memoria of an outstanding microbiologist and friend, Pierre Cornelis

**DOI:** 10.1111/1751-7915.14440

**Published:** 2024-03-27

**Authors:** Sylvie Chevalier, Olivier Lesouhaitier, Isabelle J. Schalk

**Affiliations:** ^1^ Unité de Recherche Communication Bactérienne et Stratégies Anti‐infectieuses, CBSA UR4312 Universite de Rouen Normandie Mont‐Saint‐Aignan France; ^2^ Ecole Superieure de Biotechnologie de Strasbourg, UMR7242, University of Strasbourg Illkirch France

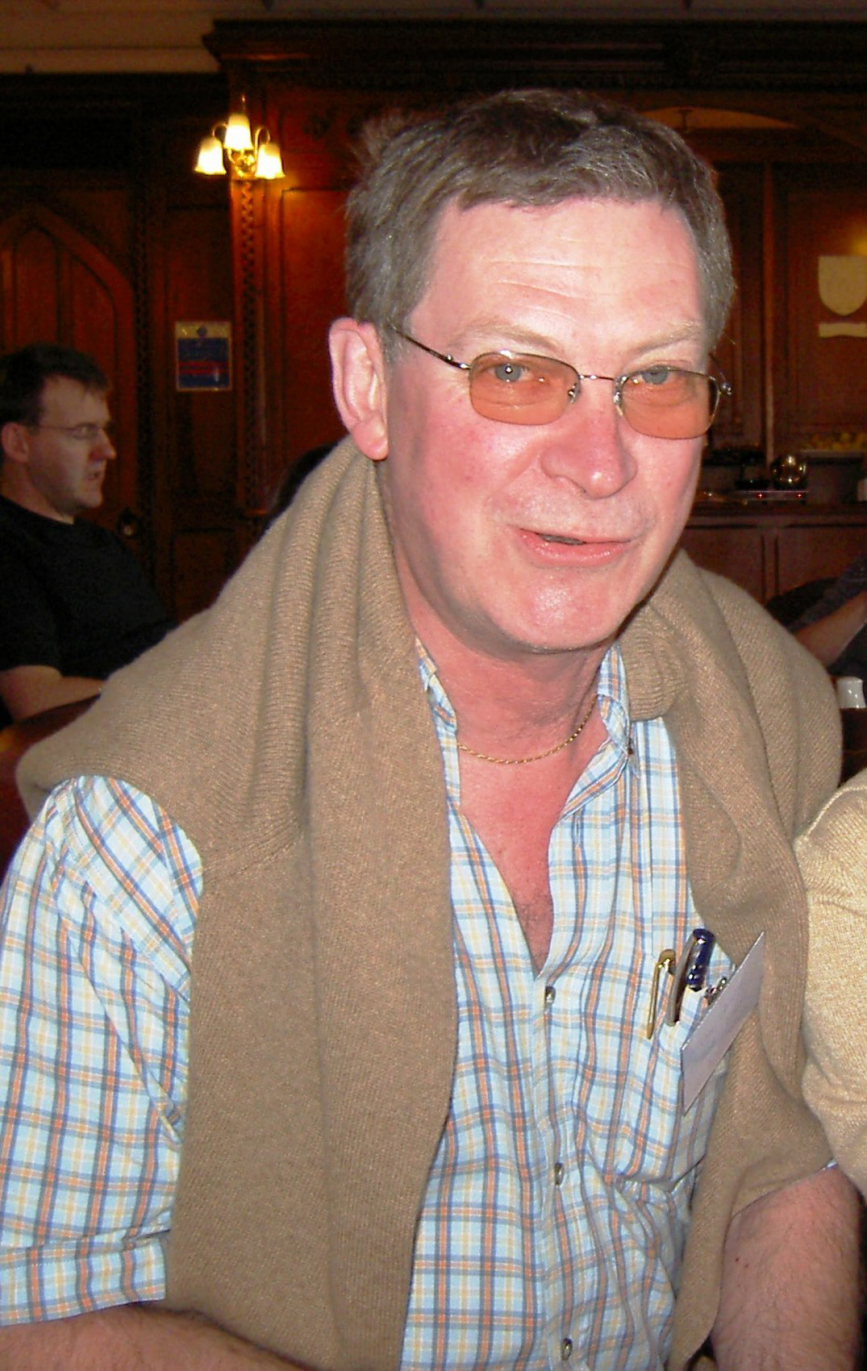

It is with profound sadness that we announce the passing of Pierre Cornelis on 2 December 2023. Born in Kinshasa, Democratic Republic of Congo, on 4 November 1949, Pierre Cornelis left an indelible mark on the scientific community through his remarkable academic achievements and groundbreaking research in microbiology, especially on *Pseudomonas*.

Pierre commenced his academic journey by completing a doctoral thesis on isoaccepting tRNA species from healthy and crown gall tobacco tissues at the Catholic University of Louvain (Belgium). His pursuit of knowledge led him to a postdoctoral stay at the Weizmann Institute of Science in Rehovot, Israel. His interest in bacterial iron homeostasis began in 1985 during his tenure at the Institute of Cellular Pathology (ICP‐UCL) in Brussels. Subsequently, he spent the majority of his career at the Vrije Universiteit Brussel. As a professor at Vrije Universiteit Brussel, Pierre shared his extensive knowledge with students in microbiology and biology, leaving a positive impact on the education of numerous generations. In recognition of his outstanding contributions, Dr. Pierre was honoured as Prof. Emeritus in October 2014.

P. Cornelis dedicated his scientific career to *Pseudomonas*, with a particular focus on the iron uptake pathways of these bacteria, the molecular mechanisms involved in the killing of *Pseudomonas* cells by pyocins, cell–cell communication in *Pseudomonas*, and gene regulation by environmental signals.

## ISABELLE J. SCHALK, UNIVERSITY OF STRASBOURG (FRANCE), COLLEAGUE IN THE FIELD OF BACTERIAL IRON HOMEOSTASIS

P. Cornelis' research provided comprehensive insights into the diversity, evolution and functionality of siderophores and their corresponding outer membrane transporters in various *Pseudomonas* species, contributing to the understanding of iron acquisition mechanisms and their implications in microbial competition and pathogenicity. Indeed, iron is a key nutrient for bacteria, paradoxically poorly bioavailable because it is poorly soluble under aerobic conditions. To access iron, most bacteria produce siderophores, small molecules with a very high affinity for iron (Hider & Kong, 2011). After scavenging iron in the bacterial environment, the ferrisiderophore complexes are recognized and imported across the outer membrane by TonB‐dependent outer membrane transporters (TBDTs) in Gram‐negative bacteria (Schalk et al., 2012). Pyoverdines are a family of siderophores produced by fluorescent Pseudomonads, giving a typical yellow‐green colour to *Pseudomonas* cultures (Ravel & Cornelis, 2003). These siderophores have peptide chains that are quite diverse, and more than 50 pyoverdine structures have been elucidated.

With his research team and colleagues, Pierre identified and contributed to the structure determination of new pyoverdines, such as the ones produced by *Pseudomonas entomophila* or *Pseudomonas putida* W15Oct28 (Budzikiewicz et al., 2007; Matthijs et al., 2009; Ye et al., 2013). He also identified new siderophores, different from pyoverdines, such as ornicorrugatin from *Pseudomonas fluorescens* AF76 (Matthijs et al., 2008) and quinolobactin, a siderophore of *Pseudomonas fluorescens* ATCC 17400 (Mossialos et al., 2000). He investigated the distribution and evolution of ferripyoverdine transporters in *Pseudomonas aeruginosa* genomes, highlighting the species' complexity with three subgroups characterized by the production of three specific pyoverdines (PVDI, PVDII and PVDIII) with different chemical structures and their corresponding TBDTs (FpvAI, FpvAII and FpvAIII; de Chial et al., 2003). Additionally, he analysed genomes of different *Pseudomonas* strains to extract the genes coding for TBDTs, gaining more insights into this family of proteins (Cornelis & Bodilis, 2009; Ye et al., 2014). For example, using multiplex PCR, he analysed the *fpvA*I, *fpvA*II, *fpvA*III genes in 345 *P. aeruginosa* strains from environmental or clinical origin, finding a similar proportion of each type in clinical strains, while FpvA type I was slightly over‐represented (49%) in environmental strains (Bodilis et al., 2009). The study suggests a complex evolutionary history linked to iron acquisition's ecological and virulent role in *P. aeruginosa*. He also identified FpvB, an alternative type I ferripyoverdine TBDT in *P. aeruginosa* and PirA a TBDT involved in iron uptake by catechol siderophores like enterobactin (Ghysels et al., 2004, 2005). This *fpvB* gene is present in the vast majority of *P. aeruginosa* strains (93%). Moreover, in the analysis of the genome of *P. fluorescens* ATCC 17400, P. Cornelis and his colleagues identified 55 TBDTs, marking the largest number of such transporters reported for *Pseudomonas* to date (Ye et al., 2014). Among these TBDTs, 15 were predicted to be ferripyoverdine transporters, highlighting that *Pseudomonas* can utilize pyoverdine produced by other *Pseudomonas* species.

Pierre and his colleagues have also investigated the mechanisms of action of various pyocins produced by *P. aeruginosa* strains under different growth conditions. Soluble (S‐type) pyocins are *P. aeruginosa* bacteriocins that kill nonimmune *P. aeruginosa* cells by gaining entry via a specific receptor at the cell surface. They first demonstrated that the uptake of pyocin S3 occurs through FpvAII (Baysse et al., 1999), the TBDT of ferripyoverdine type II of *P. aeruginosa*, and that Pyocins S2 and S4 utilize the FpvA type I ferripyoverdine transporter (Denayer et al., 2007). Pierre Cornelis and his team have shown that the N‐terminal receptor‐binding domain of pyocin S2 competes with pyocin S4 for binding to the FpvAI transporter (Elfarash et al., 2012). Moreover, they identified the gene encoding the immunity protein of pyocin S4, and its deletion renders strains sensitive to pyocin S4 (Elfarash et al., 2012). Concerning pyocin S5, they also demonstrated that this bacteriocin utilizes the FptA ferripyochelin TBDT to kill *P. aeruginosa* (Elfarash et al., 2014). Transposon mutants with insertions in the *fptA* gene, encoding the transporter for siderophore pyochelin, exhibit resistance to pyocin S5. The TBDT‐binding domain of pyocin S5 is identified as amino acid residues 151–300, not at the N‐terminus domain like for other S‐type pyocins (Elfarash et al., 2014). Pierre Cornelis and his colleagues have also described a new nuclease bacteriocin, pyocin S6, encoded in the genome of a *P. aeruginosa* cystic fibrosis clinical isolate (Dingemans et al., 2016). They demonstrated that the pyocin S6 receptor‐binding and translocation domains are identical to those of pyocin S1, whereas the killing domain is similar to the 16S ribonuclease domain of *Escherichia coli* colicin E3. They also showed that purified pyocin S6 inhibits one‐fifth of the 110 *P. aeruginosa* cystic fibrosis clinical isolates tested, showing clearer inhibition zones when the target cells are grown under iron limitation. Overall, all these findings contributed to the understanding of pyocin diversity, receptor specificity and the interplay between pyocins and the iron acquisition systems in *P. aeruginosa* under different environmental conditions.

## SYLVIE CHEVALIER AND OLIVIER LESOUHAITIER, COLLEAGUES AT THE UNIVERSITY OF ROUEN NORMANDY (FRANCE)

In 2014, Pierre Cornelis retired from the Vrije Universiteit of Brussels but continued his research activities as an Emeritus Professor. From 2014 to 2023, he served as an associate collaborator at the Laboratory of Microbiology ‘Bacterial Communication and Anti infectious Strategies’ (CBSA UR4312, former ‘Laboratory of Microbiology Signals and Environment, LMSM EA4312) at the University of Rouen Normandy (France). Through numerous enriching discussions and multiple visits to the CBSA Lab, Pierre Cornelis participated in all projects and instilled new research ideas, sharing his knowledge with others. This fruitful collaboration led to the publication of 21 articles focusing mostly on porins and their regulation in *P. aeruginosa*, and on the effect of host peptide hormones on the physiology of *P. aeruginosa*.

This strong collaboration began in 2004, stemming from discussions about OprF, the major outer membrane structural protein, which led to the discovery that OprF is involved in *P. aeruginosa* virulence (Fito‐Boncompte et al., 2011) and biofilm formation (Bouffartigues et al., 2020). It also led to a review synthetizing all the knowledge on *P. aeruginosa* porins at structural and regulatory levels (Chevalier et al., 2017). Pierre Cornelis contributed to deciphering the roles of the extracytoplasmic function sigma factor SigX in biofilm formation and virulence expression (Gicquel et al., 2013), membrane fluidity homeostasis (Fléchard et al., 2018), response to membrane‐active components (Azuama et al., 2020; Tahrioui et al., 2020), Pf4 filamentous phage infection (Tortuel et al., 2020, 2022) and temperature variations (Bouffartigues et al., 2020). These studies led to synthesizing the current knowledge on *P. aeruginosa* extracytoplasmic function sigma factors (Chevalier et al., 2019). Altogether, these studies led to proposing SigX as a new member of the cell envelope stress response (Chevalier et al., 2022). Since the envelope forms a barrier between the cell and the environment, several studies have been conducted to deepen the understanding of the molecular mechanisms affecting the outer membrane, in relation to biofilm formation and the production of virulence factors. This was the case for several phthalates and derivatives (Louis, Tahrioui, et al., 2022), and the aminoglycoside tobramycin at sub‐MIC concentrations that enhanced biofilm formation, with alterations in extracellular DNA, quorum sensing and PrrF1/F2 small RNA production (Tahrioui et al., 2019). Lastly, we showed that SigX and membrane fluidity homeostasis are key players in the biofilm increase in response to sub‐Mics of tobramycin (David et al, 2024 in Microbiology Spectrum).

He also immensely aided us in understanding the mechanism of action behind the antibiofilm effects of our molecules of interest, particularly the hormone C‐type natriuretic peptides. Pierre allowed us to make rapid progress on the mechanism of action of these peptides, thanks to his extraordinary knowledge of all the genes and metabolic pathways active in *P. aeruginosa* (Clamens et al., 2017; Lesouhaitier et al., 2019). More recently, he provided excellent guidance and hypothesized on the dispersive impact of biofilms induced by the family of natriuretic peptides such as Atrial Natriuretic peptide (ANP; Louis, Clamens, et al., 2022) and Osteocrin (Louis et al., 2023).

Pierre remained actively involved in the scientific community until his passing. In a recent event on 9 October 2023, Pierre Cornelis inaugurated the conference of the French National Network on Pseudomonas held in Mittelwihr, near Strasbourg (France). He delivered a talk focusing on future perspectives in microbiology, particularly emphasizing the field of iron homeostasis. Two weeks before his passing, we were engaged in discussing a review on the role of transcriptional antiterminators in the physiology of *P. aeruginosa*. This demonstrated, unequivocally, his commitment to scientific endeavours until the end of his life.

Cornelis Pierre's influence reached far beyond the academic realm. He served on evaluation committees for prestigious organizations, represented Vrije Universiteit Brussel at Fonds voor Wetenschappelijk Onderzoek and held leadership roles in scientific societies. His editorial roles, including Editor‐in‐Chief of Microbiology Open, Editor for the journal *Pathogens and Disease* and Editor for Biometals, as well as being a Member of the Editorial Board of Environmental Microbiology, demonstrated his commitment to advancing microbiological research. Additionally, he held the position of president of the International Biometals Society (IBS) from 2014 to 2018. He organized the 8th International Biometals Symposium held in Brussels, Belgium in 2012 and the *Pseudomonas* 2001 meeting in Brussels. Furthermore, as the Ambassador of the American Society for Microbiology (ASM) for Belgium, Pierre continued to bridge the international scientific community.

Throughout his distinguished career, Pierre wielded significant influence as a professor, researcher and editor. However, we remember Cornelis Pierre not only for his scholarly and academic achievements but also for his kindness, humility, freedom of thought and spirit, humour and dedication to the betterment of humanity through science. He was a real gentleman. We will all miss Pierre but may it bring comfort knowing that it was a tremendous privilege to have known him. May his legacy continue to inspire scientific generations to come.

## AUTHOR CONTRIBUTIONS

**Sylvie Chevalier:** Writing–review editing. **Olivier Lesouhaitier:** Writing–review editing. **Isabelle J. Schalk:** Writing–review editing.

## FUNDING INFORMATION

No funding information provided.

## CONFLICT OF INTEREST STATEMENT

The authors present no conflict of interest.

## Data Availability

Data sharing is not applicable to this article as no new data were created or analyzed in this study.
